# Senescent cell accumulation is associated with T-cell imbalance in the skin

**DOI:** 10.18699/vjgb-25-118

**Published:** 2025-12

**Authors:** K.S. Matveeva, S.K. Kolmykov, T.S. Sokolova, D.R. Salimov, D.V. Shevyrev

**Affiliations:** Sirius University of Science and Technology, Sirius Federal Territory, Krasnodar region, Russia; Sirius University of Science and Technology, Sirius Federal Territory, Krasnodar region, Russia; Sirius University of Science and Technology, Sirius Federal Territory, Krasnodar region, Russia; Sirius University of Science and Technology, Sirius Federal Territory, Krasnodar region, Russia; Sirius University of Science and Technology, Sirius Federal Territory, Krasnodar region, Russia

**Keywords:** senescence, adaptive immunity, regulatory T cells, single-cell transcriptome, aging, genetic signatures, tissue-resident T cells, senescent cell elimination, skin, сенесцентность, адаптивный иммунитет, регуляторные T-лимфоциты, транскриптом единичных клеток, старение, генетические сигнатуры, тканерезидентные T-лимфоциты, элиминация сенесцентных клеток, кожа

## Abstract

Organismal aging is accompanied by the accumulation of senescent cells – damaged, non-functional cells that exhibit cell cycle arrest, resistance to apoptosis, metabolic dysfunction, and production of a wide range of pro-inflammatory substances. The age-related accumulation of these cells is associated with impaired tissue function, contributes to chronic inflammation (inflammaging), and promotes the development of various age-associated diseases. Conversely, the elimination of senescent cells restores tissue functions and positively affects overall metabolism. Under normal conditions, senescent cells are removed by the innate immune system; however, the efficiency of this process declines with age. The involvement of adaptive immunity and the role of T cells in the clearance of senescent cells remain poorly understood. The aim of this study was to identify alterations in local T cell immunity associated with the accumulation of senescent cells in human skin. The analysis was performed on publicly available single-cell RNA-sequencing data from skin biopsies, and the senescent status was assessed using the SenePy algorithm with Gaussian mixture models. It was found that the emergence of senescent cells occurs heterogeneously across cell types within the tissue. The accumulation of these cells is associated with alterations in the CD4+ to CD8+ T cell ratio, as well as with an increased abundance of regulatory T cells. Functional analysis revealed that these quantitative age-related shifts were accompanied by more pronounced activation of regulatory T cells together with features of anergy and exhaustion in CD8+ T cells, whereas functional changes in CD4+ T cells were heterogeneous. These findings underscore the importance of adaptive immunity in maintaining tissue homeostasis and suggest potential age-related dysfunction of tissue-resident T cells. Understanding the mechanisms underlying the interaction between adaptive immunity and senescent cells is crucial for the development of senolytic vaccines and other immunological approaches aimed at enhancing endogenous elimination of senescent cells.

## Abstract (ru)

Старение организма сопровождается накоплением поврежденных нефункциональных клеток, которые называют сенесцентными. Эти клетки находятся в состоянии ареста клеточного цикла, устойчивы к апоптозу, имеют нарушенный метаболизм, а также продуцируют широкий спектр провоспалительных факторов – цитокинов, хемокинов, протеаз, молекул адгезии и продуктов арахидонового каскада. Накопление таких клеток с возрастом связано с нарушением функций тканей, способствует хроническому воспалению (inflammaging) и развитию различных возраст-ассоциированных заболеваний. В свою очередь, элиминация сенесцентных клеток восстанавливает тканевые функции и позитивно сказывается на общем метаболизме. В норме сенесцентные клетки удаляются системой врожденного иммунитета, однако с возрастом эффективность этого процесса падает. При этом участие адаптивного иммунитета и роль T-лимфоцитов в удалении сенесцентных клеток остаются неизученными. Целью исследования был поиск изменений в локальном T-клеточном иммунитете, которые связаны с накоплением сенесцентных клеток в коже человека. Анализ проводился на открытых данных РНК секвенирования единичных клеток биоптатов кожи. Сенесцентный статус клеток оценивали при помощи алгоритма SenePy с применением смешанных гауссовских моделей. Было выявлено, что появление клеток с выраженными признаками сенесцентности в пределах ткани происходит неравномерно среди клеточных типов. Накопление этих клеток ассоциировано с изменением соотношения популяций CD4+ и CD8+ лимфоцитов, а также сопряжено с увеличением содержания регуляторных Т-лимфоцитов. В ходе функционального анализа обнаружено, что данные количественные изменения с возрастом сопровождаются более выраженной активацией регуляторных Т-лимфоцитов совместно с анергией и истощением CD8+ лимфоцитов, тогда как функциональные изменения CD4+ лимфоцитов имеют гетерогенный характер. Полученные результаты подчеркивают значение адаптивного иммунитета в поддержании тканевого гомеостаза и указывают на потенциальную дисфункцию эффекторных тканевых T-лимфоцитов, которая возникает с возрастом. Понимание механизмов взаимодействия адаптивного иммунитета с сенесцентными клетками важно в контексте разработки сенолитических вакцин и других иммунологических подходов, направленных на усиление эндогенной элиминации сенесцентных клеток.

## Introduction

Cellular senescence is a state of irreversible cell cycle arrest triggered by diverse stressors, including replicative exhaustion, DNA damage, telomere shortening, oxidative stress, and oncogene activation (Regulski, 2017; Di Micco et al., 2021). Senescent cells exhibit resistance to apoptosis, diminished cellular function, metabolic dysregulation, and multiple aberrations in protein quality control machinery. A hallmark feature of these cells is their sustained secretion of a broad array of pro-inflammatory mediators, collectively termed the senescence-associated secretory phenotype (SASP). The SASP is widely regarded as a primary driver of chronic, low-grade inflammation associated with aging, commonly referred to as inflammaging. Although senescence serves as an important tumor-suppressive mechanism, the prolonged persistence and accumulation of senescent cells in tissues disrupt tissue homeostasis, impair organ function, and contribute to the pathogenesis of age-related and degenera- tive diseases (Di Micco et al., 2021; Liao et al., 2021; Witham et al., 2023).

Preclinical studies in animal models have demonstrated that targeted elimination of senescent cells improves tissue function and metabolism, extends healthspan and lifespan, and attenuates the progression of age-associated pathologies (Yousefzadeh et al., 2019; Yang et al., 2023). Under physiological conditions, senescent cells are efficiently cleared by the immune system, with innate immune mechanisms being the most extensively characterized in this context. Natural killer (NK) cells recognize senescent cells primarily via the activating receptor NKG2D and eliminate them through perforin– granzyme-mediated cytotoxicity and interferon-gamma (IFN-γ) secretion (Antonangeli et al., 2019). Invariant natural killer T (iNKT) cells can also target senescent cells upon activation by glycolipid antigens (Arora et al., 2021). Furthermore, SASP-derived factors recruit macrophages, which contribute to the clearance of senescent cells during tissue remodeling (Song P. et al., 2020). However, with advancing age, the immune system’s capacity to eliminate senescent cells declines – likely due to immunosenescence – resulting in increased senescent cell burden, chronic inflammation, tissue dysfunction, and heightened susceptibility to age-related diseases (Song S. et al., 2020; Hense et al., 2024).

Despite extensive research into the physiological clearance of senescent cells, the role of adaptive immunity in their elimination remains poorly understood (Matveeva et al., 2024). Conventional experimental approaches often inadequately reproduce the complex three-dimensional tissue architecture essential for critical interactions between adaptive immune system and senescent cells. A substantial proportion of T lymphocytes resides in peripheral tissues, does not recirculate, and exhibits functional properties distinct from those of circulating peripheral T cells (Li et al., 2025). Conversely, senescent cells are predominantly localized within the parenchyma and stroma of organs, where they can shape a unique microenvironment that modulates the efficacy of immune surveillance (Zhang W. et al., 2024). In this context, single-cell RNA sequencing (scRNA-seq) data derived directly from tissues hold particular significance. Such data enable the identification of senescent cells across diverse cell types and facilitate the assessment of key features of adaptive immunity, including the composition of specific T-cell subsets and their functional competence. By preserving the native tissue context, scRNA-seq datasets from multiple organs allow for the correlation of senescent cell burden with both quantitative and qualitative alterations in T-lymphocyte populations – the principal effectors of adaptive immunity (Kim S., Kim C., 2021).

In this study, we utilized publicly available scRNA-seq data to evaluate whether age-related accumulation of senescent cells in tissues is associated with alterations in the tissueresident T-cell pool. It is currently accepted that cellular senescence manifests differently across distinct cell types (Cohn et al., 2023). Moreover, robust and universal molecular markers of senescence applicable to all senescent cell types remain elusive. Consequently, we employed the SenePy algorithm to infer cellular senescence status. Unlike conventional differential expression analyses, SenePy identifies co-expression gene network clusters associated with aging (Sanborn et al., 2025). Skin aging is a multifaceted process driven by cumulative exposure to diverse damaging factors throughout life. Key hallmarks of skin aging include the accumulation of senescent cells, disruption of dermal extracellular matrix architecture, degradation of elastic fibers, and impairment of barrier function (Shin et al., 2025). In the present study, the identification of senescent cells within each human skin cell type, combined with quantification of various T-lymphocyte subpopulations, revealed significant age-related alterations in tissue-resident T cells that were associated with the accumulation of senescent cells

## Materials and methods

For this analysis, we used publicly available single-cell RNA sequencing (scRNA-seq) datasets deposited in the NCBI Gene Expression Omnibus (GEO) and the Genome Sequence Archive for Human (GSA-Human). Skin biopsy samples from healthy donors (n = 32; age range: 18–76 years) were automatically retrieved from these repositories (see Supplementary Materials, Table S1)1.


Supplementary Materials are available in the online version of the paper: https://vavilovj-icg.ru/download/pict-2025-29/appx42.zip


Unique Molecular Identifier (UMI) count matrices were generated from raw sequencing reads using the 10x Genomics Cell Ranger pipeline (v9.0.1). Subsequent processing of count matrices and associated metadata was primarily performed using the Scanpy toolkit (Wolf et al., 2018). Prior to downstream analysis, low-quality cells were filtered out based on the following criteria: (i) total UMI counts <500 or >5 median absolute deviations (MAD); (ii) number of detected genes >5 MAD; and (iii) mitochondrial gene expression >15 % or >4 MAD from the median. Doublets were identified and removed using the Scrublet package (Wolock et al., 2019).

**Fig. 1.**
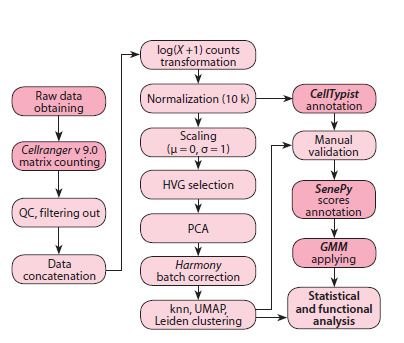
Schematic representation of the data processing workflow

Following quality control, samples were integrated into a unified dataset and prepared for clustering. This preprocessing pipeline included: (i) library-size normalization to a target sum of 10,000 UMIs per cell (scanpy.pp.normalize_total(target_ sum=1e4)); (ii) log-transformation; (iii) scaling; (iv) dimensionality reduction via principal component analysis (PCA); and (v) batch-effect correction using the Harmony algorithm (Korsunsky et al., 2019). Cell-type annotation was performed on log-normalized data using CellTypist (Domínguez et al., 2022), which employs pre-trained logistic regression models. Specifically, we applied the “Adult_Human_Skin” model (Reynolds et al., 2021), which encompasses annotations for diverse dermal, epidermal, and immune cell populations in human skin. To validate and refine automated annotations, cells were further clustered using the Leiden algorithm. Cluster identities were cross-referenced with CellTypist predictions, and manual curation of annotations was performed where necessary. The full data processing workflow is illustrated in Figure 1. Particular attention was devoted to the accurate annotation of T-lymphocyte subpopulations. To this end, the T-cell cluster was isolated from the integrated dataset and reprocessed starting from the original UMI count matrix to ensure a more precise representation of T-cell heterogeneity in reduced-dimensional space. Annotations were refined as needed based on this focused re-analysis. Samples exhibiting insufficient representation of specific cell types were excluded from relevant downstream analyses at corresponding stages of the study

Canonical markers of cellular senescence are highly cell type-specific and poorly reflect the true senescent state in vivo. Therefore, cellular senescence status was assessed using the SenePy algorithm, published in 2025 (Sanborn et al., 2025), which enables discrimination between bona fide senescenceassociated markers and genes, the expression of which is elevated for reasons unrelated to senescence. Within this algorithm, the identification of genes potentially associated with age-related accumulation of senescent cells is performed under the following criteria: the gene must be expressed in fewer than 5 % of cells from young donors, and in more than 1 % but fewer than 20 % of cells from older donors. Additionally, either the proportion of cells expressing the gene in aged individuals must be at least 2.5-fold higher than in young individuals, or the absolute increase in the proportion of expressing cells (i. e., the difference between old and young donors) must exceed 5 %. This strategy enables the identification of cell type-specific genetic signatures of senescence within a given tissue, thereby allowing more accurate detection of senescent cells in ex vivo samples compared to conventional approaches. Each cell is assigned a continuous numerical metric – the “SenePy score” – reflecting the degree to which its gene expression profile aligns with the corresponding cell type-specific senescence signature

Following SenePy scoring, Gaussian Mixture Models (GMMs) were fitted to the distribution of SenePy scores within each annotated cell type. Depending on the shape of the score distribution, models comprised either two or three components. The threshold for classifying a cell as senescent was defined as the value lying between the two rightmost GMM components. This approach enabled a quantitative estimation of the fraction of cells exhibiting robust senescence features within each cell population

Correlation analyses were performed using the spearmanr() function from the scipy.stats module to compute Spearman’s rank correlation coefficient and associated p-values. To account for multiple comparisons, Bonferroni correction was applied.

Differentially expressed genes (DEGs) in T-lymphocyte populations from young and old donors were identified using the rank_genes_groups() function from the Scanpy package, employing the Mann–Whitney U test. Genes were considered differentially expressed if they met the following criteria: false discovery rate (FDR) < 0.01, presence in more than 10 % of cells within the target group, and detection in fewer than elevated for reasons unrelated to senescence. Within this algorithm, the identification of genes potentially associated with age-related accumulation of senescent cells is performed under the following criteria: the gene must be expressed in fewer than 5 % of cells from young donors, and in more than 1 % but fewer than 20 % of cells from older donors. Additionally, either the proportion of cells expressing the gene in aged individuals must be at least 2.5-fold higher than in young individuals, or the absolute increase in the proportion of expressing cells (i. e., the difference between old and young donors) must exceed 5 %. This strategy enables the identification of cell type-specific genetic signatures of senescence within a given tissue, thereby allowing more accurate detection of senescent cells in ex vivo samples compared to conventional approaches. Each cell is assigned a continuous numerical metric – the “SenePy score” – reflecting the degree to which its gene expression profile aligns with the corresponding cell type-specific senescence signature.

Following SenePy scoring, Gaussian Mixture Models (GMMs) were fitted to the distribution of SenePy scores within each annotated cell type. Depending on the shape of the score distribution, models comprised either two or three components. The threshold for classifying a cell as senescent was defined as the value lying between the two rightmost GMM components. This approach enabled a quantitative estimation of the fraction of cells exhibiting robust senescence features within each cell population.

Correlation analyses were performed using the spearmanr() function from the scipy.stats module to compute Spearman’s rank correlation coefficient and associated p-values. To account for multiple comparisons, Bonferroni correction was applied.

Differentially expressed genes (DEGs) in T-lymphocyte populations from young and old donors were identified using the rank_genes_groups() function from the Scanpy package, employing the Mann–Whitney U test. Genes were considered differentially expressed if they met the following criteria: false discovery rate (FDR) <0.01, presence in more than 10 % of cells within the target group, and detection in fewer than 50 % of cells in the comparison group. Functional enrichment analysis of the identified DEGs was performed in the R programming language using the enricher() function from the clusterProfiler package (Yu et al., 2021). Gene sets from the C5 (ontology gene sets) and C7 (immunologic signature gene sets) collections of the Molecular Signatures Database (MSigDB; Subramanian et al., 2005) were used as reference annotations. Significantly enriched gene sets were manually grouped into functional categories.

## Results

**Fig. 2.**
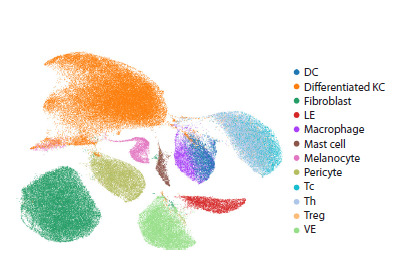
Cell type annotation of human skin using the CellTypist tool. DC – dendritic cells; KC – keratinocytes; LE – lymphoid epithelial cells; Tc – cytotoxic T lymphocytes (classical phenotype: CD3+CD8+); Th – T helper cells (classical phenotype: CD3+CD4+); Treg – regulatory T cells (classical phenotype: CD3+CD4+FoxP3+); VE – vascular endothelial cells.

To identify senescent cells in human skin tissues, we adapted and applied the recently published SenePy algorithm (Sanborn et al., 2025), followed by Gaussian Mixture Modeling (GMM). The analysis was performed on the major skin cell populations previously annotated (Fig. 2).

**Fig. 3.**
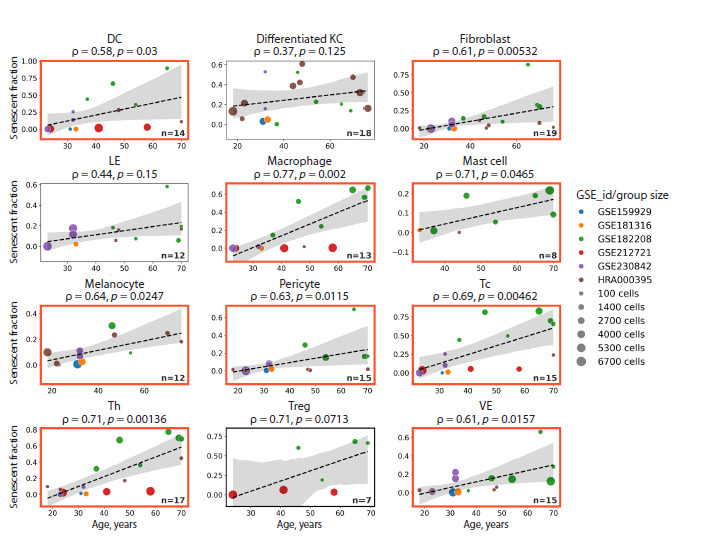
Correlations between the accumulation of senescent cells in distinct human skin cell types and donor age. For each cell type, samples with cell counts below 2SD (standard deviations) from the mean across all donors were excluded from the analysis. Statistically significant correlations are highlighted with red boxes. DC – dendritic cells; KC – keratinocytes; LE – lymphoid epithelial cells; Tc – cytotoxic T lymphocytes; Th – T helper cells; Treg – regulatory T cells; VE – vascular endothelial cells.

As a result, we observed a significant age-associated increase in the proportion of senescent cells across multiple cell types in human skin samples (Fig. 3). Specifically, the fraction of senescent cells rose with age in tissue-resident dendritic cells, macrophages, T lymphocytes, keratinocytes, melanocytes, fibroblasts, pericytes, and endothelial cells. Notably, the rate of accumulation varied between cell types, reflecting the heterogeneity of aging processes among distinct cellular populations within the same tissue.

**Fig. 4.**
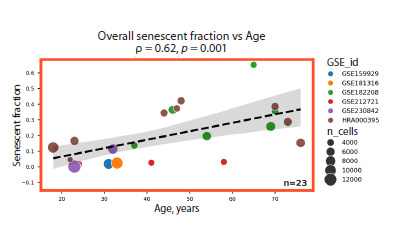
Proportion of senescent cells across all cell types as a function of donor age.

Our analysis revealed a significant age-related accumulation of cells exhibiting senescence features in the skin, consistent with prior evidence implicating cellular senescence as a key hallmark of tissue aging (Childs et al., 2015). The overall proportion of senescent cells across all cell types also showed a positive correlation with donor age (Fig. 4), indicating a progressive disruption of tissue homeostasis. Given that senescent cells are characterized by a stable cell cycle arrest and thus lack proliferative capacity, their age-dependent accumulation is likely attributable to a decline in the efficiency of mechanisms responsible for their clearance.

**Fig. 5.**
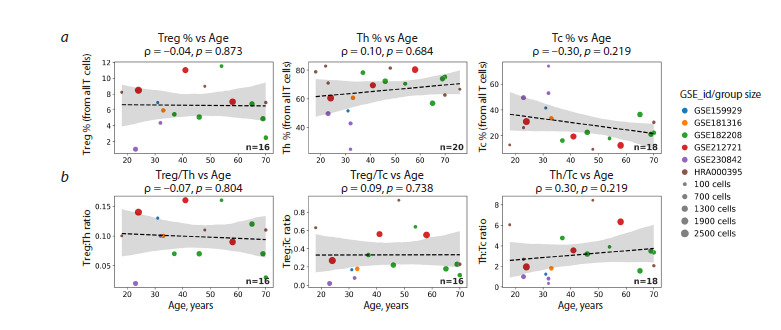
Age-related changes in the proportions of major T-lymphocyte populations (a) and their ratios (b). The immunological indices shown – Th/Tc, Treg/Tc, and Treg/Th ratios – are widely used to assess immune status with greater precision and sensitivity in various pathological or compromised conditions. In this figure, the proportion of each T-lymphocyte subset is expressed relative to the total number of T lymphocytes, thereby reflecting the balance among subpopulations within the entire pool of skin-resident T cells. Treg – regulatory T cells; Th – T helper cells; Tc – cytotoxic T lymphocytes.

Therefore, in the next step, we sought to investigate how the proportions of major T-lymphocyte subpopulations in the skin change with age. Correlation analysis did not reveal statistically significant age-related changes in the proportions of the three T-lymphocyte subpopulations examined, nor in key immunological indices (Fig. 5). Given the absence of detectable age-associated alterations among tissue-resident T lymphocytes, we next sought to explore potential associations between T-lymphocyte populations and the accumulation of senescent cells independent of chronological age.

Different cell types may exhibit varying rates of aging or differing immunogenicity of their senescent counterparts, which could account for the observed heterogeneity in age-related accumulation of senescent cells. Therefore, we first sought to determine whether any alterations in skin T-lymphocyte populations were associated with the burden of senescent cells. Specifically, we assessed the relationship between the accumulation of senescent cells within each cell type and the relative abundance of T-lymphocyte subpopulations (Fig. S1). We found a significant increase in total T-lymphocyte frequency associated with the accumulation of senescent pericytes, as well as modest trends (p < 0.07) toward elevated regulatory T-cell (Treg) proportions correlating with senescent cell burden in certain cell types.

**Fig. 6.**
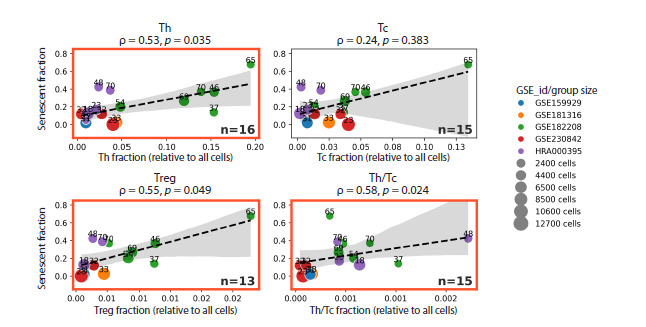
Proportions of major T-lymphocyte populations relative to the total number of senescent cells. In this figure, the abundance of each T-lymphocyte subset is expressed as a fraction of the total cell count across all cell types, rather than as a proportion of the total T-cell pool. This approach captures age-independent shifts in T-lymphocyte representation within the entire skin cellular landscape and more accurately reflects biologically relevant changes associated with the accumulation of senescent cells. Th – T helper cells; Tc – cytotoxic T lymphocytes; Treg – regulatory T cells.

In the next step, we examined how the proportions of different T-lymphocyte populations vary with the total burden of senescent cells across all cell types. We observed a significant increase in the relative abundance of both T helper (Th) cells and regulatory T (Treg) cells as the cumulative number of senescent cells rose (Fig. 6). Moreover, we noted a statistically significant elevation in the “tissue immunoregulatory index” – defined as the Th/Tc ratio – which reflects a shift toward T helper dominance over cytotoxic T lymphocytes.

Thus, we identified a significant association between the accumulation of senescent cells in human skin and an imbalance in T-cell immunity. This imbalance was characterized by an increased proportion of regulatory T cells and T helper cells, accompanied by a relative decrease in cytotoxic T lymphocytes. Notably, these alterations were not directly correlated with chronological age, underscoring the specific role of interactions between T-cell immunity and senescent cells, independent of aging per se.

**Fig. 7.**
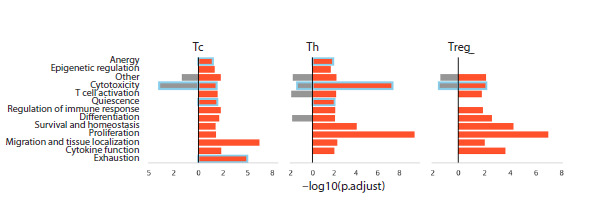
Results of functional enrichment analysis of differentially expressed genes (DEGs) in tissue-resident T-lymphocyte populations from older versus younger donors. Red bars represent enrichment of functional pathways by upregulated genes, while gray bars indicate enrichment by downregulated genes. The X-axis shows the –log10-transformed FDR-corrected p-value, such that higher values correspond to stronger enrichment. Tc – cytotoxic T lymphocytes; Th – T helper cells; Treg – regulatory T cells.

The age-independent shifts in the tissue-resident T-lymphocyte pool observed in earlier analyses highlight the involvement of adaptive immunity in tissue aging processes. However, these findings do not provide insight into the functional states of Treg cells, Th, or cytotoxic T lymphocytes. To further characterize the functional implications of these changes, we performed differential gene expression analysis followed by functional enrichment profiling of T-lymphocyte populations (see Materials and methods), comparing cells from older versus younger donors (Fig. 7)

Functional enrichment analysis revealed statistically significant overrepresentation of biological pathways associated with enhanced functional activity of T helper (Th) cells, including tissue adaptation, differentiation, and response to cytokines involved in their homeostasis. Additionally, enrichment of pathways characteristic of quiescent and anergic states was observed in this population (highlighted with blue boxes). Notably, however, these Th cells did not exhibit clear molecular signatures of exhaustion. In contrast, age-related alterations in cytotoxic T lymphocytes were associated with enrichment of pathways typical of quiescence, anergy, and exhaustion. Intriguingly, this Tc population also displayed significant downregulation of pathways directly linked to their effector function – particularly cytotoxicity. Conversely, regulatory T cells showed no evidence of quiescence, anergy, or exhaustion. Instead, similar to Th cells, Treg cells exhibited heightened functional and proliferative activity. Moreover, this population demonstrated significant enrichment of genes involved in differentiation and response to homeostatic cytokines – specifically IL-2, IL-7, and IL-15 – which are essential for the maintenance and survival of tissue-resident regulatory T cells (Table S2).

Thus, functional enrichment analysis of differentially expressed genes (DEGs) identified from scRNA-seq data revealed distinct functional states across T-lymphocyte subsets. Cytotoxic T lymphocytes exhibited clear signatures of exhaustion and reduced functional activity. In contrast, regulatory T cells displayed heightened functional activity and showed no evidence of exhaustion or anergy. Changes in the Th population were more heterogeneous: alongside increased functional activity, these cells also exhibited features characteristic of anergy and quiescence.

## Discussion

The accumulation of senescent cells is a hallmark of tissue aging and is closely linked to the development of chronic, low-grade systemic inflammation – termed “inflammaging” – which constitutes a major risk factor for age-related diseases (Franceschi et al., 2018). Using a modern algorithm for identifying senescence-associated gene signatures, we demonstrated that the proportion of cells exhibiting senescence features increases with age in human skin. Importantly, this accumulation is not uniform across all cell types, underscoring the heterogeneity of aging trajectories among distinct cellular populations and highlighting the multifaceted nature of tissue aging (Ge et al., 2022).

The immune system plays a central role in the surveillance and clearance of senescent cells. The pro-inflammatory secretome of senescent cells – commonly referred to as the senescence-associated secretory phenotype (SASP) – recruits innate immune effectors such as macrophages, neutrophils, natural killer (NK) cells, and NKT cells, which contribute to the recognition and elimination of senescent cells (Song P. et al., 2020). Although emerging evidence implicates T lymphocytes in these processes, the role of adaptive immunity in senescent cell clearance remains incompletely understood (Matveeva et al., 2024). Our findings reveal that the burden of senescent cells in human skin is associated with a local imbalance in T-cell immunity, suggesting that T lymphocytes actively participate in regulating senescent cell homeostasis. Notably, higher senescent cell loads correlated with an increased proportion of regulatory T cells and an elevated Th/Tc ratio. This shift points toward the establishment of an immunosuppressive microenvironment that may facilitate immune evasion by senescent cells (Zhang W. et al., 2024). This interpretation is further supported by functional profiling of T-cell populations in older donors. Cytotoxic T lymphocytes exhibited molecular signatures of exhaustion and diminished effector potential, whereas both Treg and Th cells displayed heightened functional activity and signs of tissue adaptation. Collectively, these quantitative and qualitative alterations in the skin-resident T-cell compartment in aged individuals may promote peripheral tolerance to senescence-associated antigens. This aligns with the hypothesis that aging impairs the immune system’s capacity to recognize and efficiently eliminate senescent cells, thereby contributing to their progressive accumulation (Song P. et al., 2020).

It is well established that senescent cells not only generate a pro-inflammatory milieu but also can actively suppress effector T-cell functions and evade immune surveillance (Lorenzo et al., 2022). For instance, certain SASP-derived chemokines selectively recruit Treg-cells, while senescence-driven polarization of monocytes toward an M2-like macrophage phenotype suppresses cytotoxic T-cell activation (Zhang X. et al., 2024). Moreover, aging-associated activation of endogenous retroelements – particularly LINE-1 – triggers an IFN-γ- mediated response (Zhang X. et al., 2020). This antiviral-like response may fuel chronic inflammation and drive T-cell exhaustion, a phenotype strikingly reminiscent of the cytotoxic T-cell dysfunction observed in our cohort of older donors.

In summary, our data indicate that the skin T-cell compartment undergoes substantial functional remodeling with age. The decline in cytotoxic activity coupled with enhanced regulatory T-cell function may foster immunological tolerance, thereby enabling the persistence and accumulation of senescent cells and contributing to inflammaging. We propose that this represents an active process of peripheral tolerance to senescence-associated antigens, wherein the aging immune system progressively loses its ability to detect and eliminate senescent cells. The identified imbalance in tissue-resident T-lymphocyte populations thus constitutes a promising therapeutic target for interventions aimed at restoring immune surveillance and promoting the clearance of senescent cells.

## Conclusion

In this study, we employed bioinformatic analyses of publicly available scRNA-seq data derived from skin biopsies of healthy donors to identify aging-associated alterations in tissue-resident adaptive immunity. We demonstrated that skin aging – manifested as the accumulation of senescent cells across multiple cell types – is associated with a shift in the balance between Th and cytotoxic T lymphocytes, as well as an increased proportion of Treg cells. Functional enrichment analysis further revealed a general decline in cytotoxic potential among tissue T cells, concurrent with enhanced regulatory activity. These changes likely reflect compensatory adaptations within the tissue T-cell compartment in response to the persistent accumulation of senescent cells and the resulting chronic inflammatory microenvironment. In this context, the observed T-cell remodeling appears to promote an immunosuppressive milieu, potentially contributing to the age-related decline in the efficiency of senescent cell clearance.

scRNA-seq data provide a powerful tool for investigating immune-senescence interactions at the tissue level. Preservation of the tissue cellular context enables the identification of physiologically relevant aging signatures and facilitates the analysis of gene programs associated with activation or suppression of specific immune components. Nevertheless, this approach has inherent limitations. The loss of spatial tissue architecture precludes direct assessment of cell-to-cell interactions, while technical artifacts introduced during sample preparation and data integration from multiple sources necessitate rigorous preprocessing, batch-effect correction, and normalization – steps that may introduce substantial uncertainty into the results. Therefore, to gain a deeper understanding of the role of adaptive immunity in the surveillance and elimination of senescent cells, future studies should integrate scRNA-seq with spatial transcriptomics, histological validation, and methods capable of defining the antigen specificity of T and B cells. Additionally, longitudinal analyses of T- and B-cell receptor repertoires will be essential to elucidate dynamic changes in antigen recognition during aging and their functional consequences for immune-mediated clearance of senescent cells.

## Conflict of interest

The authors declare no conflict of interest.
